# Clinical Characterization of Patients With 5q Spinal Muscular Atrophy Types 2 and 3 in Brazil: A Cross‐Sectional Observational Study

**DOI:** 10.1111/cge.70176

**Published:** 2026-05-08

**Authors:** Elice Carneiro Batista, Edmar Zanoteli, Henrique Andrade R. Fonseca, Graziela Jorge Polido, Jonas Alex Morales Saute, Adriana Banzzatto Ortega, Marcondes Cavalcante França Junior, Acary Souza Bulle Oliveira, Paulo Victor Sgobbi de Souza, Juliana Gurgel Giannetti, André Luiz Santos Pessoa, Alexandra Prufer de Queiroz Campos Araújo, Mário Fritsch Toros Neves, Raquel Tavares Boy da Silva, Frederico Monfardini, Gustavo Prado dos Santos, Bruna dos Santos Sampaio, Diogo Fagundes Moia, Fernando Galan Júnior, Luciana Pereira Almeida de Piano, Camila Santos Nascimento de Albuquerque, Flávia Miranda Duarte, Viviane Aparecida Rodrigues Sant’Anna, Mariane Pereira, Jomenica de Bortoli Livramento, Ronaldo Vicente Pereira Soares, Denison Alves Pedrosa, Vanessa Teich, Luiz Vicente Rizzo, Otávio Berwanger, João Brainer Clares de Andrade, Gisele Sampaio Silva

**Affiliations:** ^1^ Hospital Israelita Albert Einstein São Paulo Brazil; ^2^ Departamento de Neurologia, Faculdade de Medicina Universidade de São Paulo (FMUSP) São Paulo São Paulo Brazil; ^3^ Faculdade de Medicina, Hospital de Clínicas de Porto Alegre, Serviços de Genética Médica e de Neurologia, Universidade Federal do Rio Grande do Sul Porto Alegre Rio Grande do Sul Brazil; ^4^ Hospital Infantil Pequeno Príncipe – Curitiba Curitiba Brazil; ^5^ Departamento de Neurologia, Faculdade de Ciências Médicas Universidade Estadual de Campinas Campinas São Paulo Brazil; ^6^ Departamento de Neurologia e Neurocirurgia Universidade Federal de São Paulo São Paulo São Paulo Brazil; ^7^ Departamento de Pediatria, Faculdade de Medicina Universidade Federal de Minas Gerais Belo Horizonte Minas Gerais Brazil; ^8^ Hospital Infantil Albert Sabin (HIAS) Fortaleza Brazil; ^9^ Departamento de Pediatria, Faculdade de Medicina Universidade Federal do Rio de Janeiro Rio de Janeiro Rio de Janeiro Brazil; ^10^ Hospital Universitário Pedro Ernesto (HUPE) Rio de Janeiro Brazil; ^11^ Imperial College London London UK; ^12^ George Institute for Global Health London UK

**Keywords:** clinical registry, disease‐modifying treatment, rare disease, SMA, spinal muscular atrophy

## Abstract

Spinal muscular atrophy (SMA), linked to chromosome 5q, is a rare hereditary neurodegenerative disease characterized by progressive motor neuron loss. It is classified into subtypes based on age at onset and the highest motor milestone achieved. This cross‐sectional observational study aimed to describe the clinical profile of patients with types 2 and 3 SMA followed within the Brazilian Public Health System (SUS). Clinical data from patients with types 2 and 3 SMA followed at nine national reference centers (2020–2021) were analyzed. A total of 155 patients were included: 76 with type 2 and 79 with type 3 SMA. Disease duration was longer in type 3 patients. Time from symptom onset to genetic confirmation was also longer in this group. Functional impairment was observed in both subtypes. Type 2 patients had lower HFMSE scores overall, though higher scores were seen among those on disease‐modifying therapies. In type 3, earlier symptom onset and longer disease duration were associated with worse motor outcomes. HFMSE scores varied with treatment use and disease duration. Findings reveal the clinical heterogeneity of SMA and emphasize the impact of diagnostic delays and disease duration on function. Early diagnosis and ongoing multidisciplinary care are crucial.

## Graphical abstract

This study describes the clinical heterogeneity of Brazilian patients with 5q spinal muscular atrophy types 2 and 3, highlighting prolonged diagnostic delays and the impact of disease duration on motor function. Early genetic diagnosis and access to multidisciplinary care are crucial to preserve functional outcomes.

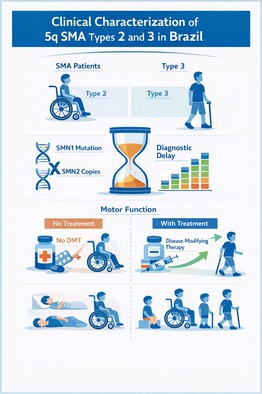

## 1 Introduction

Spinal muscular atrophy linked to chromosome 5q (SMA or 5q SMA) is an autosomal recessive hereditary neuromuscular disease characterized by the degeneration of motor neurons in the spinal cord and brain stem, resulting in progressive muscle weakness [1, 2]. The estimated incidence of SMA is around 1 case per 10 000 live births, and the prevalence is one to two cases per 100 000 individuals [3].

The disease is caused by homozygous deletions or loss‐of‐function mutations in the *SMN1* gene at the 5q13 locus, leading to insufficient expression of the survival motor neuron (SMN) protein [1, 4, 5, 6, 7, 8, 9, 10]. The classification of 5q SMA is based on the age of symptom onset and the highest motor milestone achieved [1, 2, 5, 7, 10, 11]. Type 1 SMA manifests by 6 months of age with an inability to sit without support, type 2 between 6 and 18 months with an inability to walk independently, and type 3 after 18 months with an initial ability to walk, which may be lost over time [1, 7, 12]. Type 4, with adult‐onset, reflects a broader phenotypic spectrum without clear subtype delineations [11, 12, 13].

The main genetically determining factor of the clinical variability of the disease is the copy number of the *SMN2*, an *SMN1*‐homologous gene [14]. Advances in understanding 5q SMA pathogenesis prompted the emergence of promising therapeutic strategies, resulting in several clinical trials being performed worldwide [15, 16, 17, 18, 19, 20].

Effective SMN‐dependent therapies, such as gene therapy with *SMN1* gene replacement (onasemnogene abeparvovec) and splicing regulation of exon 7 in *SMN2* (nusinersen, risdiplam), have already been approved by the leading international regulatory agencies and the Brazilian National Surveillance Agency (ANVISA) [21]. While nusinersen and risdiplam are approved for use in all clinical forms of SMA‐5q, gene therapy is approved for children up to 2 years of age. Several pivotal and real‐world studies have demonstrated the efficacy of these therapies in different clinical forms of 5q‐SMA [21, 22, 23, 24]. These studies have emphasized the importance of early initiation of therapies, as the earlier treatment starts, the better the outcome [21]. Children diagnosed and treated in the pre‐symptomatic phase tend to achieve near‐normal motor function. This underscores the urgent need for effective newborn screening programs [25]. In addition, many studies, including Brazilian patients, have demonstrated that there is still a significant delay for the diagnosis, especially for late‐onset forms of the disease [24]. In Brazil, the Unified Health System (SUS) offers, within a public policy program, access to three DMTs for SMA: nusinersen for types 1 and 2, risdiplam for types 1 and 2, and Onasemnogene abeparvovek for type 1 in the first 6 months of life. Type 3 patients do not have access to DMT in SUS, needing to file lawsuits for access to DMT, or follow treatment with DMT in private healthcare or supplementary healthcare. A deeper understanding of the epidemiology, the medication's effects, and temporal milestones could be crucial in planning its integration, designing clinical protocols, and potentially expanding access to therapy. Therefore, the present cross‐sectional study aimed to characterize the clinical condition of patients with 5q SMA types 2 and 3 in Brazil.

## 2 Methods

### 2.1 Study Design and Participants

**FIGURE 1:**
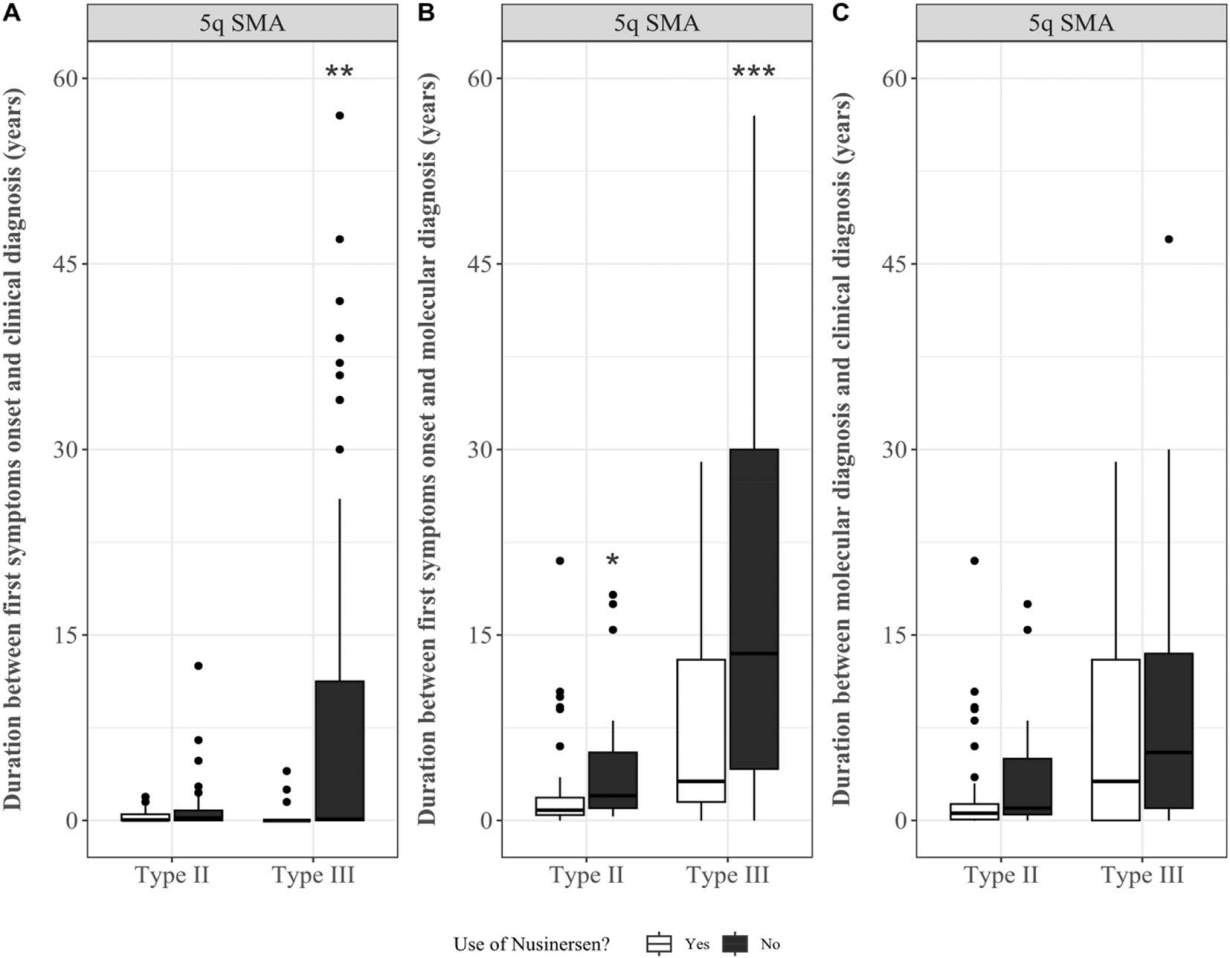
Time elapsed between the appearance of the first signs and symptoms until confirmation of the diagnosis by the molecular report. (A) Interval between the onset of the first signs and symptoms and the clinical diagnosis of patients with 5q SMA types 2 and 3. (B) Interval between the onset of the first signs and symptoms and the molecular diagnosis of patients with 5q SMA types 2 and 3. (C) Interval between clinical diagnosis and confirmation by molecular report in patients with 5q‐SMA types 2 and 3. *p value = 0.01146 (Mann–Whitney *U*‐test); 5q SMA type 2 patients using nusinersen versus 5q SMA type 2 patients without using nusinersen. **p value = 0.002056 (Mann–Whitney *U*‐test); 5q SMA type 3 patients using nusinersen versus 5q SMA type 3 patients without using nusinersen. ***p value = 0.01329 (Mann–Whitney *U*‐test); 5q SMA type 3 patients using nusinersen versus 5q SMA type 3 patients without using nusinersen.

**FIGURE 2:**
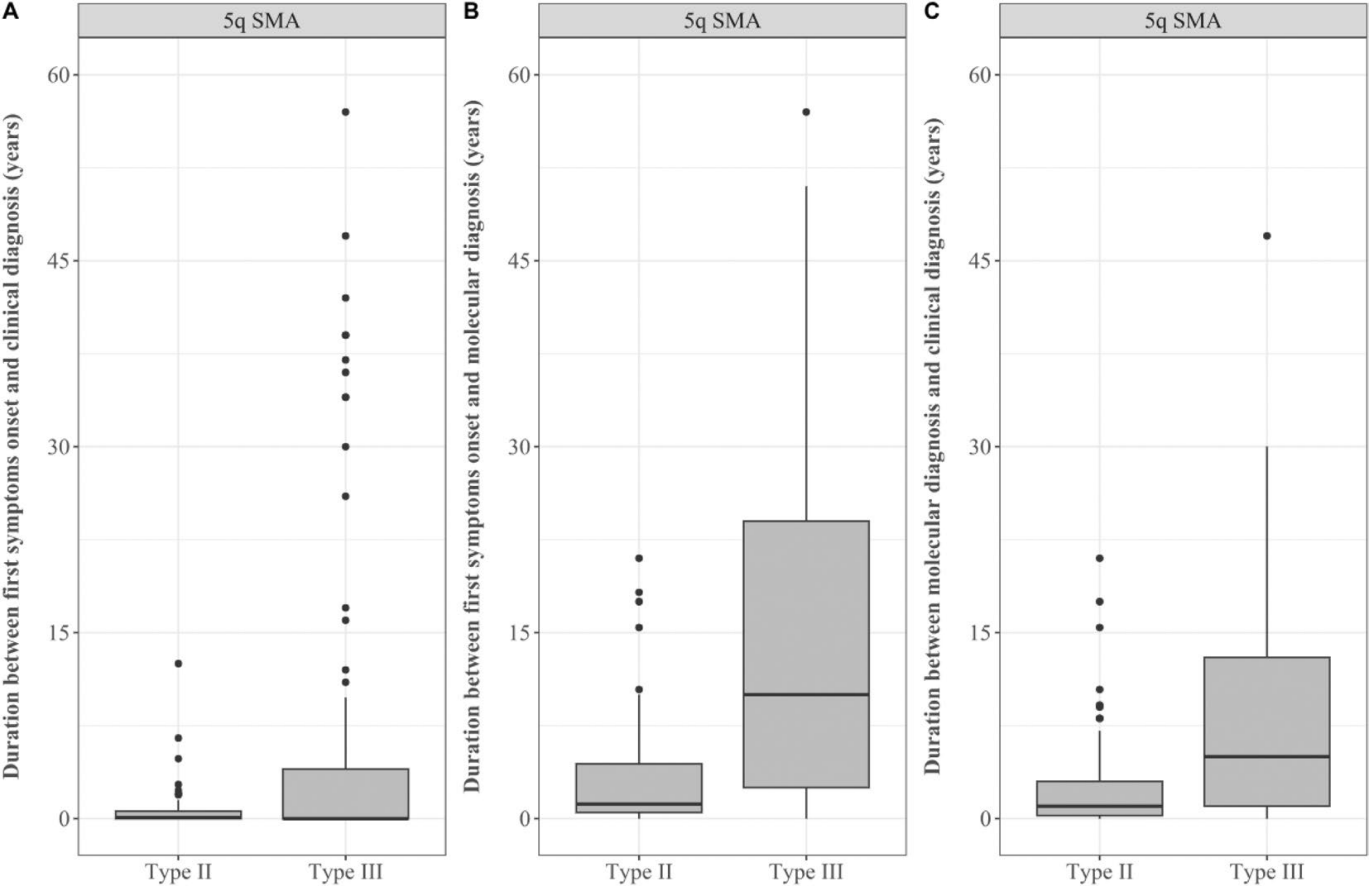
No separation of patients with or without nusinersen use: Time elapsed between the appearance of the first signs and symptoms until confirmation of the diagnosis by the molecular report. (A) Interval between the onset of the first signs and symptoms and the clinical diagnosis of patients with 5q SMA types 2 and 3. (B) Interval between the onset of the first signs and symptoms and the molecular diagnosis of patients with 5q SMA types 2 and 3. (C) Interval between clinical diagnosis and confirmation by molecular report in patients with 5q SMA types 2 and 3.

This observational study (ClinicalTrials.gov: NCT04404764) analyzed clinical data from patients with 5q SMA types 2 and 3 who were followed at nine national Brazilian reference neurological centers from 2020 to 2021 (Figures 1 and 2). Patients of all ages and both sexes were included. The Ethics Committees of each institution approved the study, and a complete list of inclusion and exclusion criteria is presented in Table 1.

**TABLE 1:** Study enrolment criteria.

Inclusion criteria
Participants of both sexes, in any age group, who have a clinical diagnosis of 5q SMA types 2 and 3, in follow‐up at SUS, undergoing treatment with nusinersen or not;
2 Clinical and molecular diagnosis of 5q SMA type 2 (disease started after 6 months of age), or Clinical and molecular diagnosis of 5q SMA type 3 (disease started after 18 months of age).

Exclusion criteria
Refusal to provide written informed consent (either the patient or a legal representative);
2 Symptom onset after 19 years of age;
3 Need for invasive ventilatory support for 16 h or more per day for more than 21 consecutive days;
4 Be participating or have participated in another clinical study aimed at the specific treatment of 5q SMA other than with the drug nusinersen;
5 Having undergone treatment with gene therapy.

### 2.2 Procedures

All potentially eligible individuals who visited study sites were requested to undergo an SMA genetic test to analyze the *SMN1* gene. Genetic tests were performed in certified laboratories to investigate mutations in exon 7 of the *SMN1* gene. After providing written informed consent, eligible participants were recruited by the centers to evaluate clinical history and medical record data obtained during the clinical visit and follow‐up. Genetic confirmation was based on molecular testing performed at certified laboratories. However, due to the real‐world nature of this study and historical variability in diagnostic practices in Brazil, detailed characterization of SMN1 variants and SMN2 copy number was not consistently available. In some cases, genetic reports often performed several years prior to study inclusion reported findings compatible with 5q SMA without detailed specification of the variant. All included patients had a clinically confirmed diagnosis established by experienced neuromuscular specialists at reference centers.

### 2.3 Motor Scores Outcome

Motor scores were recorded if participants had assessments from the primary scales used to evaluate clinical conditions, such as the Hammersmith Functional Motor Scale Expanded (HFMSE), the Revised Upper Limb Module (RULM), and the Children's Hospital of Philadelphia Infant Test of Neuromuscular Disorders (CHOP‐INTEND). Local investigators experienced in applying these scales were responsible for documenting the scores.

### 2.4 Statistical Analysis

A convenience sample was used with a target of approximately 100 participants. However, this number could be increased as more patients met the inclusion criteria within the study timeline. Clinical motor endpoints included the HFMSE score at baseline. Clinical characteristics were described for the two subtypes and stratified by participants with and without access to DMT. Quantitative variables were presented as means and standard deviations for normally distributed data or as medians and interquartile ranges for non‐normally distributed data. Qualitative variables were expressed as absolute frequencies (number of patients) and relative frequencies (percentages). Treatment exposure was heterogeneous, particularly regarding duration of nusinersen use. Given the cross‐sectional design of the study, no attempt was made to standardize treatment duration or to formally assess treatment response over time. The first signs and symptoms of the disease were analyzed based on the time between the genetic report confirming 5q SMA and the signs documented in the medical records. The HFMSE score was evaluated as the disease progressed using the Dunn test and the Kruskal–Wallis test.

Data were entered into the web‐based electronic case report form (e‐CRF) system, REDCap, and IBM. The system's functionalities included patient registration, data entry, data cleaning, and export for statistical analysis.

### 2.5 Role of the Funding Source

The study was designed and led by an academic steering committee and sponsored by a Brazilian Ministry of Health grant. The Ministry had no role in study design, data collection, site selection, data management, statistical analysis, or the decision to publish the manuscript (Brazilian Ministry of Health—PROADI‐SUS, grant number NUP‐25000.165213/2020‐69).

## 3 Results

We included a total of 155 participants: 76 patients diagnosed with SMA type 2 and 79 with SMA type 3 (Table 2). Participants' ages ranged from 30 months to 72 years (SD ±17.08 years). Among type 2 patients, 39.5% (30/76) were female, while in the type 3 group, 49.4% (39/79) were female. Disease duration varied significantly between groups. The mean disease duration for SMA type 2 was 8.90 years (SD ±7.05), whereas for SMA type 3, it was 18.62 years (SD ±15.41). At the time of the study, 39 patients with type 2 and 21 with type 3 were on the use of nusinersen.

**TABLE 2:** Profile of 155 patients with 5q SMA types 2 and 3 being monitored in the Brazilian Unified National Health System (SUS).

	5q SMA type 2 (*N* = 76)	5q SMA type 3 (*N* = 79)	Total (*N* = 155)
Sex			
Female	30 (39.5%)	39 (49.4%)	69 (44.5%)
Male	46 (60.5%)	40 (50.6%)	86 (55.5%)
Age (years)			
Mean (SD)	10.08 (7.17)	23.81 (18.45)	17.08 (15.65)
Median (Q1, Q3)	8.37 (4.70, 12.60)	19.16 (8.77, 35.08)	10.87 (6.17, 21.26)
Min–Max	1.70–43.61	3.05–72.53	1.70–72.53
Type of mutation			
Deletion in heterozygosity	4 (5.3%)	13 (16.5%)	17 (11.0%)
Homozygous deletion	72 (94.7%)	66 (83.5%)	138 (89.0%)
Number of copies of the SMN2 gene			
1	1 (1.3%)	4 (5.1%)	5 (3.2%)
2	9 (11.8%)	3 (3.8%)	12 (7.7%)
3	45 (59.2%)	44 (55.7%)	89 (57.4%)
4 or more	5 (6.6%)	13 (16.5%)	18 (11.6%)
Unknown	16 (21.1%)	15 (19.0%)	31 (20.0%)
Disease duration (years)			
Mean (SD)	8.90 (7.05)	18.62 (15.41)	13.86 (12.97)
Median (Q1, Q3)	7.00 (4.00, 12.25)	16.00 (7.00, 25.00)	9.00 (5.00, 18.00)
Min–Max	0.50–42.00	0.33–65.00	0.33–65.00
Motor function			
Head control			
Doesn't hold his head up	11 (14.5%)	2 (2.5%)	13 (8.4%)
Head kept in a vertical position at all times	55 (72.4%)	56 (70.9%)	111 (71.6%)
Unknown	10 (13.2%)	21 (26.6%)	31 (20.0%)
Can sit without support			
Unable to sit without support	4 (5.3%)	0 (0.0%)	4 (2.6%)
Sit with support	26 (34.2%)	24 (30.4%)	50 (32.3%)
Sit without support	34 (44.7%)	34 (43.0%)	68 (43.9%)
Unknown	12 (15.8%)	21 (26.6%)	33 (21.3%)
Standing			
Does not stand	55 (72.4%)	20 (25.3%)	75 (48.4%)
Stands with support	8 (10.5%)	10 (12.7%)	18 (11.6%)
Stands without support	2 (2.6%)	27 (34.2%)	29 (18.7%)
Unknown	11 (14.5%)	22 (27.8%)	33 (21.3%)
Walking ability			
Don't walk	42 (55.3%)	8 (10.1%)	50 (32.3%)
Walk with support	21 (27.6%)	22 (27.8%)	43 (27.7%)
Walking independently	1 (1.3%)	27 (34.2%)	28 (18.1%)
Unknown	12 (15.8%)	22 (27.8%)	34 (21.9%)

	5q SMA type 2 (*N* = 76)	5q SMA type 3 (*N* = 79)	Total (*N* = 155)
Deglutition			
Difficulty in deglutition	14 (18.4%)	12 (15.2%)	26 (16.8%)
No difficulty in deglutition	61 (80.3%)	62 (78.5%)	123 (79.4%)
Unknown	1 (1.3%)	5 (6.3%)	6 (3.9%)
Has a cough during or after meals			
No	65 (85.5%)	65 (82.3%)	130 (83.9%)
Yes	8 (10.5%)	6 (7.6%)	14 (9.0%)
Unknown	3 (4.0%)	8 (10.1%)	11 (7.0%)
Use a gastric or nasal tube			
No	71 (93.4%)	75 (94.9%)	146 (94.2%)
Yes	4 (5.3%)	1 (1.3%)	5 (3.2%)
Unknown	1 (1.3%)	3 (3.8%)	4 (2.6%)
Type of tube (*n* = 5 patients in use)			
Gastric tube	4 (100.0%)	1 (100.0%)	5 (100.0%)
Contractures			
No	15 (19.7%)	35 (45.5%)	50 (32.7%)
Yes	55 (72.4%)	32 (41.6%)	87 (56.9%)
Unknown	6 (7.9%)	10 (13.0%)	16 (10.5%)
Scoliosis			
No	17 (22.4%)	26 (32.9%)	43 (27.7%)
Yes	59 (77.6%)	52 (65.8%)	111 (71.6%)
Unknown	0 (0.0%)	1 (1.3%)	1 (0.6%)
Wheelchair use			
No	13 (17.1%)	33 (41.8%)	46 (29.7%)
Part‐time	11 (14.5%)	10 (12.7%)	21 (13.5%)
Full‐time	52 (68.4%)	35 (44.3%)	87 (56.1%)
Unknown	0 (0.0%)	1 (1.3%)	1 (0.6%)
Has pain regularly			
No	58 (76.3%)	53 (67.1%)	111 (71.6%)
Yes	14 (18.4%)	17 (21.5%)	31 (20.0%)
Unknown	4 (5.3%)	9 (11.4%)	13 (8.4%)
Fatigue			
No	54 (71.1%)	41 (51.9%)	95 (61.3%)
Yes	13 (17.1%)	32 (40.5%)	45 (29.0%)
Unknown	9 (11.8%)	6 (7.6%)	15 (9.7%)
Use ventilatory support			
No	49 (64.5%)	72 (91.1%)	121 (78.1%)
Yes	25 (32.9%)	6 (7.6%)	31 (20.0%)
Unknown	2 (2.6%)	1 (1.3%)	3 (1.9%)
Tracheostomy			
No	75 (98.7%)	77 (97.5%)	152 (98.1%)
Yes	1 (1.3%)	0 (0.0%)	1 (0.6%)
Unknown	0 (0.0%)	2 (2.5%)	2 (1.3%)
Changes in the electrocardiogram			
No	24 (31.6%)	27 (34.2%)	51 (32.9%)
Yes	3 (3.9%)	2 (2.5%)	5 (3.2%)
Unknown	49 (64.5%)	50 (63.3%)	99 (63.9%)
Cognition			
Amended	0 (0.0%)	2 (2.5%)	2 (1.3%)
Normal	67 (88.2%)	71 (89.9%)	138 (89.0%)
Unknown	9 (11.8%)	6 (7.6%)	15 (9.7%)
Other neurological signs			
No	57 (75.0%)	58 (73.4%)	115 (74.2%)
Yes	10 (13.2%)	16 (20.3%)	26 (16.8%)
Unknown	9 (11.8%)	5 (6.3%)	14 (9.0%)
Recent hospitalizations (last 12 months)			
No	67 (88.2%)	73 (92.4%)	140 (90.3%)
Yes	4 (5.3%)	4 (5.1%)	8 (5.2%)
Unknown	5 (6.6%)	2 (2.5%)	7 (4.5%)
Reason for recent reported hospitalizations			
Scoliosis surgery	1	1	2
Placement of a catheter for the application of nusinersena (there was an infection, treated with antibiotics)	1	0	1
Femur fracture (caused by falling from a wheelchair)	0	1	1
Urinary tract infection	1	0	1
Kidney stone lithotripsy	0	1	1
Complications due to deep vein thrombosis	0	1	1
Vomiting and dehydration	1	0	1
Associated disease			
No	61 (80.2%)	65 (82.3%)	126 (81.3%)
Yes	10 (13.2%)	11 (13.9%)	21 (13.5%)
Unknown	5 (6.6%)	3 (3.8%)	8 (5.2%)
Reported associated diseases			
Renal tubular acidosis to be clarified	1	0	1
Anxiety	1	0	1
Asthma	5	2	7
Epileptic seizures	0	1	1
Depression	1	1	2
Diabetes mellitus	0	1	1
Systemic arterial hypertension	0	4	4
Hypertrophy of adenoids and tonsils	1	0	1
Hypothyroidism	1	1	2
Renal lithiasis	1	0	1
Osteoporosis	0	2	2
Allergic rhinitis	1	0	1
Obstructive sleep apnea syndrome (severe)	0	1	1
Panic syndrome	0	1	1
Generalized anxiety disorder	0	1	1
Deep vein thrombosis	0	1	1
Use of nusinersena (disease‐modifying therapy)			
Using medication (nusinersen via legal action)	39 (51.3%)	21 (26.6%)	60 (38.7%)
Without using disease‐modifying therapy	37 (48.7%)	58 (73.4%)	95 (61.3%)

A homozygous deletion of exon 7 in the *SMN1* gene was identified in 94.7% (72/76) of type 2 patients and 83.5% (66/79) of type 3 patients. Among those who underwent molecular testing for *SMN2* copy number, 57.4% had three or more *SMN2* copies. In 20% (*n* = 31) of cases, molecular reports included only the detection of SMN1 gene mutations (Table 2).

The average time from symptom onset to genetic confirmation of 5q SMA was 3.13 years (SD ±4.45) for type 2 patients. The longest delay from symptom onset to genetic confirmation was 21 years in a type 2 patient. In type 3 patients, this interval was significantly longer, averaging 15.1 years (SD ±15.3), with the longest delay reaching 57 years (Table 3).

**TABLE 3:** Time elapsed between the appearance of the first signs and symptoms until confirmation of the diagnosis by the molecular report, considering: Interval between the onset of the first signs and symptoms and the clinical diagnosis of patients with 5q SMA types 2 and 3; Interval between the onset of the first signs and symptoms and the molecular diagnosis of patients with 5q SMA types 2 and 3; and Interval between clinical diagnosis and confirmation by molecular report in patients with 5q SMA types 2 and 3.

	5q SMA			
	Type 2		Type 3	
Use of DMT?	Yes	No	Yes	No
	(*N* = 39)	(*N* = 37)	(*N* = 21)	(*N* = 58)
Duration between molecular diagnosis and clinical diagnosis (years)				
Mean (SD)	2.16 (4.21)	2.89 (4.09)	7.55 (8.75)	8.70 (10.2)
Median [Q1, Q3]	0.50 [0.04, 1.29]	1.00 [0.42, 5.00]	3.17 [0, 13.00]	5.00 [0.25, 13.00]
[Min, max]	[0, 21.00]	[0, 17.50]	[0, 29.00]	[0, 47.00]
Missing	0 (0%)	0 (0%)	0 (0%)	0 (0%)
Duration between first symptom onset and molecular diagnosis (years)				
Mean (SD)	2.40 (4.21)	3.91 (4.61)	7.93 (8.85)	17.6 (16.4)
Median [Q1, Q3]	0.75 [0.37, 1.71]	2.00 [1.00, 5.50]	3.17 [1.50, 13.00]	13.25 [4.04, 29.25]
[Min, max]	[0, 21.00]	[0.33, 18.25]	[0, 29.00]	[0, 57.00]
Missing	0 (0%)	0 (0%)	0 (0%)	0 (0%)
Duration between first symptom onset and clinical diagnosis (years)				
Mean (SD)	0.231 (0.483)	1.02 (2.40)	0.381 (1.04)	8.94 (15.3)
Median [Q1, Q3]	0 [0, 0.42]	0 [0, 0.75]	0 [0, 0]	0 [0, 10.69]
[Min, max]	[0, 1.92]	[0, 12.50]	[0, 4.00]	[0, 57.00]
Missing	0 (0%)	0 (0%)	0 (0%)	0 (0%)

*Note*: *p = 0.01146, Mann–Whitney U test, comparing 5q SMA type 2 patients using DMT versus not using DMT. **p = 0.002056, Mann–Whitney U test, comparing 5q SMA type 3 patients using DMT versus not using DMT. ***p = 0.01329, Mann–Whitney U test, additional comparison among 5q SMA type 3 subgroups as specified in the analysis.

Abbreviation: DMT, disease‐modifying therapy (nusinersen).

### 3.1 5q SMA Type 2

**FIGURE 3:**
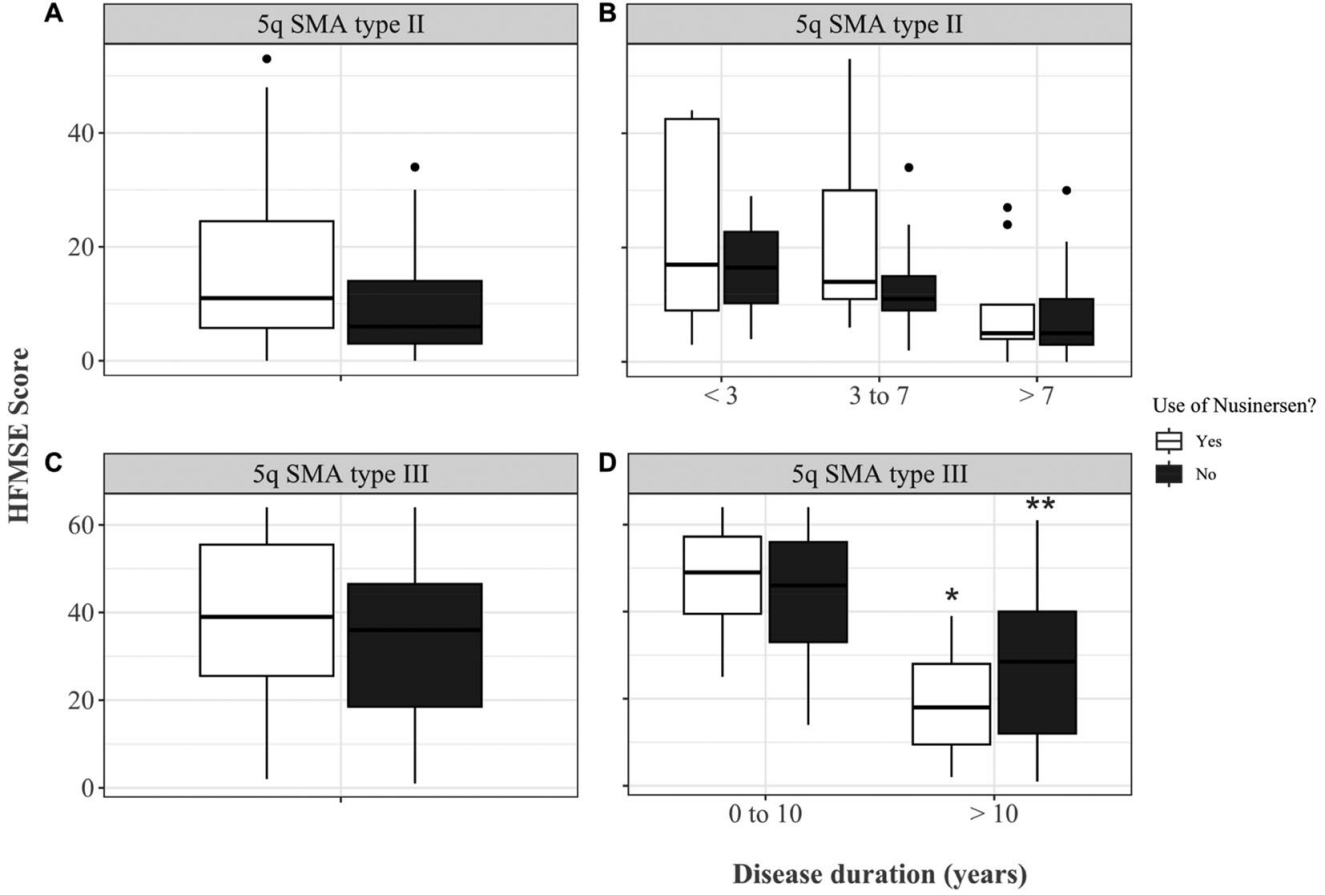
Score on the Expanded Hammersmith Functional Motor Scale (HFMSE). (A) 5q SMA type 2 patients with and without use of nusinersen. (B) 5q SMA type 2 patients with and without use of nusinersen, stratified by disease duration interval. (C) 5q SMA type 3 patients with and without use of nusinersen. (D) 5q SMA type 3 patients with and without use of nusinersen, stratified by disease duration interval. *p value = 0.006 adjusted when comparing patients with up to 10 years of time disease (who are currently using nusinersen) versus patients > 10 years of time disease (who are currently using nusinersen), Dunn's test (*p* unadjusted value = 0.001, Kruskal–Wallis test). **p value = 0.019 adjusted when comparing patients with up to 10 years of time disease (who are not using nusinersen) versus patients > 10 years of time disease (who are not using nusinersen), Dunn's test (unadjusted *p* value = 0.013, Kruskal–Wallis test).

Comparisons between treated and untreated patients should be interpreted in light of heterogeneity in treatment duration and exposure. In type 2 SMA patients, swallowing difficulties were reported in 18.4% (14/76) of the patients, with 10.5% (8/76) experiencing coughing during or after meals and 5.3% (4/76) requiring gastric tube feeding. Scoliosis was present in 77.6% (59/76) of the patients. Signs of shortening were observed in 72.4% (55/76) (*p* = 0.0001). The mean HFMSE score for type 2 patients was 14.28 (SD ±13.78). Among nusinersen users, the maximum score reached 53 points, averaging 17.16 (SD ±15.55). Non‐users' maximum score was 34 points, with a mean of 10.60 (SD ±10.04). When stratifying type 2 patients based on disease duration (< 3, 3–7, and > 7 years), a trend suggested that patients receiving nusinersen tended to achieve higher scores. However, the sample size in each stratum was small, and the difference was not statistically significant (*p* = 0.0972) (Figure 3A,B, Supporting Information 1).

### 3.2 5q SMA Type 3

The separate evaluation of intervals between symptom onset, clinical diagnosis, and molecular confirmation provides additional insight into diagnostic pathways and access to genetic testing in the Brazilian healthcare setting. Among type 3 SMA patients, swallowing difficulties were less frequent than in type 2 patients. In total, 15.2% (12/79) experienced difficulty swallowing, 7.6% (6/79) reported coughing during or after meals, and only 1.3% (1/79) required a gastric tube. Scoliosis was observed in 65.8% (52/79) of patients, with no significant difference compared to type 2 (*p* = 0.146). Signs of shortening were detected in 41.6% (32/79) of patients. Motor function assessments showed that type 3 patients had higher HFMSE scores than type 2 patients. The highest HFMSE score recorded in type 3 patients was 64 points, observed in both nusinersen users and non‐users. The mean HFMSE score among nusinersen users was 36.89 (SD ±18.71), whereas in non‐users, it was 33.64 (SD ±17.56) (Table 4). The onset of symptoms in type 3 patients varied widely, ranging from after 18 months of age to before 19 years of age. In general, patients with later‐onset symptoms exhibited better motor function. Over time, disease progression was characterized by the loss of previously acquired motor functions.

**TABLE 4:** Profile of patients with 5q SMA type 2 and type 3 monitored in the Brazilian Unified National Health System (SUS), with and without use of nusinersen (disease‐modifying therapy [DMT]).

5q SMA type 2			
	5q SMA type 2 with DMT (*N* = 39)	5q SMA type 2 without DMT (*N* = 37)	Total (*N* = 76)
Sex			
Female	13 (33.3%)	17 (45.9%)	30 (39.5%)
Male	26 (66.7%)	20 (54.1%)	46 (60.5%)
Age (years)			
Mean (SD)	8.24 (4.98)	12.03 (8.57)	10.08 (7.17)
Median (Q1, Q3)	6.47 (4.49, 10.81)	9.63 (7.02, 16.43)	8.37 (4.70, 12.60)
Min–Max	3.10–24.53	1.70–43.61	1.70–43.61
Age at clinical diagnosis			
Mean (SD)	1.15 (0.56)	1.99 (2.48)	1.56 (1.82)
Median (Q1, Q3)	1.00 (0.79, 1.38)	1.00 (0.92, 2.00)	1.00 (0.83, 1.50)
Min–Max	0.58–2.92	0.33–14.00	0.33–14.00
Age at genetic diagnosis			
Mean (SD)	3.31 (4.22)	4.88 (4.61)	4.07 (4.46)
Median (Q1, Q3)	2.00 (1.00, 2.50)	3.00 (2.00, 6.00)	2.00 (1.50, 5.25)
Min–Max	0.75–22.00	1.00–19.00	0.75–22.00
Disease duration (years)			
Mean (SD)	7.22 (5.18)	10.68 (8.31)	8.90 (7.05)
Median (Q1, Q3)	6.00 (2.92, 10.00)	8.00 (6.00, 15.00)	7.00 (4.00, 12.25)
Min–Max	0.50–23.00	1.33–42.00	0.50–42.00
Motor function			
Head control			
Doesn't hold his head up	7 (17.9%)	4 (10.8%)	11 (14.4%)
Head kept in a vertical position at all times	30 (76.9%)	25 (67.6%)	55 (72.4%)
Unknown	2 (5.1%)	8 (21.6%)	10 (13.2%)
Can sit without support			
Unable to sit without support	2 (5.1%)	2 (5.4%)	4 (5.3%)
Sit with support	17 (43.6%)	9 (24.3%)	26 (34.2%)
Sit without support	17 (43.6%)	17 (45.9%)	34 (44.7%)
Unknown	3 (7.7%)	9 (24.3%)	12 (15.8%)
Standing			
Does not stand	27 (69.2%)	28 (75.7%)	55 (72.4%)
Stands with support	7 (17.9%)	1 (2.7%)	8 (10.5%)
Stands without support	2 (5.1%)	0 (0.0%)	2 (2.6%)
Unknown	3 (7.7%)	8 (21.6%)	11 (14.5%)
Walking ability			
Stands without support	1 (2.6%)	0 (0.0%)	1 (1.3%)
Stands with support	10 (25.6%)	11 (29.7%)	21 (27.6%)
Don't walk	25 (64.1%)	17 (45.9%)	42 (55.3%)
Unknown	3 (7.7%)	9 (24.3%)	12 (15.8%)

5q SMA type 3			
	5q‐SMA with type 3 DMT (*N* = 21)	5q SMA type 3 without DMT (*N* = 58)	Total (*N* = 79)
Sex			
Female	10 (47.6%)	29 (50.0%)	39 (49.4%)
Male	11 (52.4%)	29 (50.0%)	40 (50.6%)
Age (years)			
Mean (SD)	14.50 (11.42)	27.18 (19.41)	23.81 (18.45)
Median (Q1, Q3)	9.80 (5.96, 18.34)	21.21 (10.48, 39.58)	19.16 (8.77, 35.08)
Min–Max	3.05–40.19	3.26–72.53	3.05–72.53
Age at clinical diagnosis			
Mean (SD)	4.07 (4.66)	14.28 (18.25)	11.57 (16.42)
Median (Q1, Q3)	3.00 (1.50, 4.00)	4.00 (2.00, 16.00)	3.00 (2.00, 14.00)
Min–Max	0.58–17.00	0.08–64.00	0.08–64.00
Age at genetic diagnosis			
Mean (SD)	11.62 (11.48)	22.98 (19.56)	19.96 (18.41)
Median (Q1, Q3)	4.00 (3.00, 15.00)	16.00 (6.00, 37.50)	14.00 (4.00, 33.00)
Min–Max	2.00–38.00	0.83–64.00	0.83–64.00
Disease duration (years)			
Mean (SD)	10.69 (8.90)	21.50 (16.30)	18.62 (15.41)
Median (Q1, Q3)	7.00 (4.00, 14.00)	17.50 (8.25, 30.00)	16.00 (7.00, 25.00)
Min–Max	0.50–31.00	0.33–65.00	0.33–65.00
Motor function			
Head control			
Doesn't hold his head up	0 (0.0%)	2 (3.4%)	2 (2.5%)
Head kept in a vertical position at all times	15 (71.4%)	41 (70.7%)	56 (70.9%)
Unknown	6 (28.6%)	15 (25.9%)	21 (26.6%)
Can sit without support			
Unable to sit without support	0 (0.0%)	0 (0.0%)	0 (0.0%)
Sit with support	7 (33.3%)	17 (29.3%)	24 (30.4%)
Sit without support	8 (38.1%)	26 (44.8%)	34 (43.0%)
Unknown	6 (28.6%)	15 (25.9%)	21 (26.6%)
Standing			
Does not stand	6 (28.6%)	14 (24.1%)	20 (25.3%)
Stands with support	2 (9.5%)	8 (13.8%)	10 (12.7%)
Stands without support	7 (33.3%)	20 (34.5%)	27 (34.2%)
Unknown	6 (28.6%)	16 (27.6%)	22 (27.8%)
Walking ability			
Stands without support	6 (28.6%)	16 (27.6%)	22 (27.8%)
Stands with support	8 (38.1%)	19 (32.8%)	27 (34.2%)
Don't walk	1 (4.8%)	7 (12.1%)	8 (10.1%)
Unknown	6 (28.6%)	16 (27.6%)	22 (27.8%)

Score Hammersmith Functional Motor Scale Expanded (HFMSE)			
	5q SMA type 2 (*N* = 76)	5q SMA type 3 (*N* = 79)	Total (*N* = 155)
Mean (SD)	14.28 (13.70)	34.58 (17.82)	25.17 (18.94)
Median (Q1, Q3)	9.00 (4.00, 21.00)	36.00 (20.75, 47.00)	24.00 (8.00, 40.50)
Min–Max	0.00–53.00	1.00–64.00	0.00–64.00
Unknown	19	13	32

	5q SMA type 2 with DMT (*N* = 39)	5q SMA type 2 without DMT (*N* = 37)	Total (*N* = 76)
Mean (SD)	17.16 (15.55)	10.60 (10.04)	14.28 (13.70)
Median (Q1, Q3)	11.00 (5.75, 24.50)	6.00 (3.00, 14.00)	9.00 (4.00, 21.00)
Min–Max	0.00–53.00	0.00–34.00	0.00–53.00
Mean (SD)	7	12	19

	5q SMA type 3 with DMT (*N* = 21)	5q SMA type 3 without DMT (*N* = 58)	Total (*N* = 79)
Mean (SD)	36.89 (18.71)	33.64 (17.56)	34.58 (17.82)
Median (Q1, Q3)	39.00 (25.50, 55.50)	36.00 (18.50, 46.50)	36.00 (20.75, 47.00)
Min–Max	2.00–64.00	1.00–64.00	1.00–64.00
Unknown	2	11	13

A considerable variation was noted when comparing HFMSE scores of type 3 patients with and without nusinersen. A slight trend suggested better scores among patients receiving nusinersen; however, the difference was not statistically significant (*p* = 0.5192, Mann–Whitney *U*‐test) (Figure 3C,D, Supporting Information 3). It is important to note that patients receiving nusinersen started treatment at different stages, with some having received only initial doses while others had been treated for over a year. Additional functional scales, including the revised upper limb module (RULM), were collected as part of the study; however, due to incomplete data availability across centers, these results were not included in the present analysis.

**FIGURE 4:**
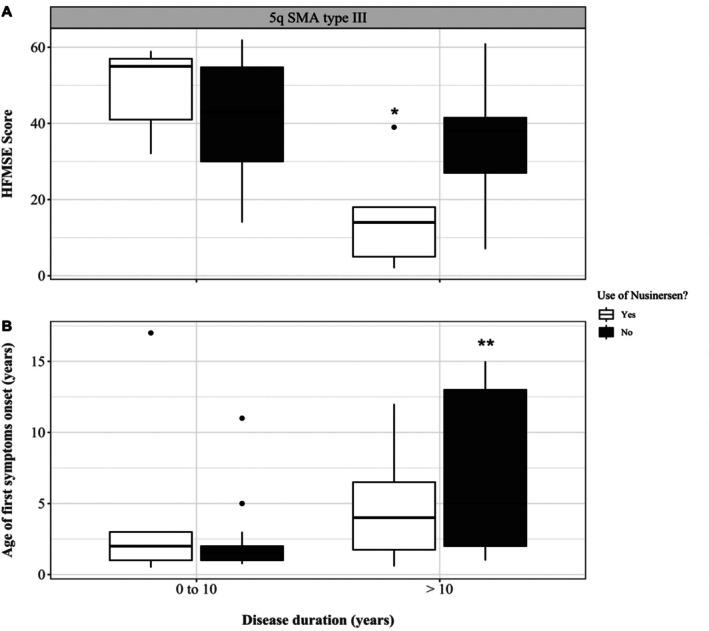
(A) Score on the Expanded Hammersmith Functional Motor Scale (HFMSE) in ambulatory 5q SMA type 3 patients (patients able to walk with support and independently): Groups stratified by disease time interval. *p value = 0.010 adjusted when comparing patients with up to 10 years of disease (who are currently using nusinersen) versus patients > 10 years of disease (who are currently using nusinersen), Dunn's test (*p* unadjusted value = 0.001, Kruskal–Wallis test). (B) Age at the onset of the first signs and symptoms in 5q SMA type 3 patients—Stratification in terms of disease duration. **p value = 0.001 adjusted when comparing patients with up to 10 years of illness (who are not currently using nusinersen) versus patients > 10 years of illness (who are currently not using nusinersen), test of Dunn.

A clear pattern of motor function loss was observed when stratifying by disease duration (≤ 10 years vs. > 10 years). Among type 3 patients using nusinersen, those with a disease duration of ≤ 10 years had significantly better motor function than those with a disease duration > 10 years (*p* = 0.006). A similar difference was found when comparing non‐users of nusinersen in the same disease duration categories (*p* = 0.019; Figure 4; Supporting Information 2). A further analysis focused only on type 3 patients who could walk (with or without support). Significant differences were found when comparing those with ≤ 10 years of disease versus those with > 10 years currently using nusinersen (*p* adjusted = 0.010; Figure 4). Likewise, differences were observed when comparing patients with ≤ 10 years of disease versus those with > 10 years who were not using nusinersen (*p* = 0.001; Figure 4).

## 4 Discussion

The 5q SMA is a rare disease that presents different clinical phenotypes or severity levels and results in high infant mortality and significant family, social, and economic impact [6, 26]. Our study mapped and analyzed the clinical profile of Brazilian patients with 5q SMA types 2 and 3 who were followed at specialized reference centers within the Brazilian Unified National Health System (SUS) during 2020 and 2021. The findings highlight the heterogeneous nature of the disease, with a wide variation in age, symptom onset, and disease duration among participants, even within the same subtype, confirming what is observed in other studies [2, 27, 28, 29, 30, 31, 32]. Motor function outcomes varied according to disease duration and access to DMT, particularly nusinersen, a single treatment available for patients with SMA. The HFMSE scores reflected the impact of disease progression, with a trend indicating better motor function preservation in patients receiving nusinersen. However, the study's cross‐sectional nature limits the ability to establish definitive trends, emphasizing the need for longitudinal follow‐up to assess disease evolution and treatment outcomes more effectively.

Our results confirmed the importance of genetic diagnosis in 5q SMA. In most cases, the disease is caused by a homozygous deletion of exon 7 of the *SMN1* gene, which was also observed in this study. However, in 20% of participants, the molecular report did not provide information on *SMN2* copy number, a limitation related to cost and the year the test was performed. The *SMN2* gene copy number is a known phenotype modifier, influencing disease severity and progression. Access to detailed genetic testing is essential for disease prognosis and therapeutic decision‐making.

The functional impairments observed in our patients were consistent with the natural history of the disease. Lower limb involvement was greater than upper limb involvement, with muscle weakness and atrophy not distributed homogeneously. The earlier the onset of symptoms and the longer the disease duration, the worse the motor outcomes. Among type 2 patients, 34 individuals achieved the milestone of sitting independently, while in the type 3 group, 27 participants could walk independently. These findings emphasize the importance of regular motor function assessments, as disease progression in types 2 and 3 is marked by the loss of previously acquired motor abilities without specialized care and DMT. The earlier the appearance of the first signs and symptoms and the longer the disease lasts, without proper specialized support, the greater the functional loss will be, according to the natural history of the disease [32, 33, 34, 35, 36, 37, 38, 39, 40, 41, 42].

The long diagnostic journey of these patients was reflected in the study's findings, where longer delays in genetic confirmation were associated with worse motor function outcomes. For type 2 patients, the average time from symptom onset to clinical diagnosis was shorter in those receiving nusinersen (0.23 years SD ±0.48) than non‐users (1.02 years SD ±2.40). Considering all type 2 patients without stratification by nusinersen use, the mean interval was 0.62 years (SD ±1.74). These findings highlight inequities in diagnostic access and treatment initiation, reinforcing the need for improved early detection strategies and genetic testing availability.

The variability in disease duration influenced HFMSE scores, reflecting the heterogeneity of disease progression. Age and motor capacity had a direct impact on motor assessment results. The HFMSE scale was chosen as the primary functional measure, as it is the most commonly used in specialized care centers. A trend was observed suggesting that type 2 patients receiving DMT had better‐preserved motor function, but the study's cross‐sectional nature prevents confirmation of this trend without longitudinal analysis. In type 3 patients, a difference in HFMSE scores was observed between those using and not using DMT. Still, this difference was more strongly correlated with disease duration rather than treatment status. Patients with an earlier onset of symptoms and a longer disease duration exhibited greater motor decline, consistent with the natural history of SMA.

Among type 3 patients who had not received treatment and had been living with the disease for over 10 years, motor function loss followed a gradual decline pattern. This group demonstrated higher HFMSE scores in earlier stages, likely due to a later onset of muscle weakness, which allowed them to achieve better motor function before disease progression. However, as time passed, further decline and potential loss of ambulation were expected, reinforcing the importance of early intervention and continuous monitoring.

Early multidisciplinary care and access to disease‐modifying therapy (DMT) are crucial to preserving motor function and slowing disease progression. Ideally, treatment should begin in the pre‐symptomatic or very early symptomatic phase. Patients with long‐standing disease and severe motor or respiratory impairment tend to show less favorable responses to treatment, as motor neuron loss is irreversible. Regardless of treatment status, long‐term follow‐up is essential, emphasizing the need for individualized therapeutic plans. Treatment strategies should be based on the patient's age, disease severity, and response to therapy, with regular motor and respiratory assessments to optimize patient outcomes. In chronic cases, motor and respiratory stabilization can be a positive outcome, particularly for individuals with advanced disease stages. Before initiating DMT, a detailed baseline assessment by a multidisciplinary team is necessary to set realistic expectations and align treatment goals with the patient's clinical condition.

Our findings reinforce the urgent need for expanded diagnostic access, earlier treatment initiation, and continuous monitoring to improve the clinical outcomes and quality of life of individuals with SMA types 2 and 3 in Brazil.

## 5 Limitations of the Study

This study has limitations. The genetic reports were not fully standardized, reflecting real‐world variability in diagnostic practices, particularly in earlier years. Detailed characterization of SMN1 variants and SMN2 copy number was not consistently available, which may limit genotype–phenotype correlations. Treatment exposure was heterogeneous, particularly regarding duration of nusinersen use, precluding formal assessment of treatment response or causal inference. Although additional functional measures such as RULM were collected, incomplete data limited their inclusion in the analysis. The absence of SMN2 copy number data in a proportion of patients represents an important limitation, as this is a key modifier of disease severity and progression. Our study has the inherent limitations of a cross‐sectional design, which does not allow for prospective analysis of patient progression over time. It is important to emphasize that the objective of this study was not to assess the effectiveness of DMTs. Future studies should focus on tracking the clinical evolution of patients over time, considering the heterogeneity of the disease in terms of duration, severity of motor impairment, and access to multidisciplinary care and DMTs. This study was conducted during the COVID‐19 pandemic. This period saw fewer hospitalizations, which does not reflect the usual healthcare routine for these patients but rather the challenges in accessing medical services during the pandemic. Acknowledging the limitations of this study, a new longitudinal follow‐up study of 5q SMA patients has been proposed, expanding beyond Brazil to include other Latin American countries (LATAM RegistrAME [43]).

## 6 Conclusion

Our study provides a comprehensive overview of the clinical profile of Brazilian patients with SMA types 2 and 3, highlighting the heterogeneity in disease progression and motor function. Our findings emphasize the prolonged time to genetic diagnosis, the impact of disease duration on functional abilities, and the need for early and continuous multidisciplinary care. The observed differences in motor function between patients with and without access to DMT reinforce the importance of timely intervention and structured follow‐up. Given the challenges in diagnosis and treatment access, particularly in resource‐limited settings, further longitudinal studies are essential to understand disease progression better and optimize patient care strategies.

## Author Contributions

Writing – protocol final version and draft manuscript, visualization, methodology, investigation, formal analysis, data curation, project administration, funding acquisition, and conceptualization: Elice Carneiro Batista. Writing – draft manuscript review and editing, supervision, methodology, and funding acquisition: Edmar Zanoteli and Henrique Andrade R. Fonseca. Draft manuscript revision and methodology: Graziela Jorge Polido. Original protocol and draft manuscript revision, methodology, and data curation: Jonas Alex Morales Saute, Adriana Banzzatto Ortega, Marcondes Cavalcante França Junior, Acary Souza Bulle Oliveira, Paulo Victor Sgobbi de Souza, Juliana Gurgel Giannetti, André Luiz Santos Pessoa, Alexandra Prufer de Queiroz Campos Araújo, Mário Fritsch Toros Neves, and Raquel Tavares Boy da Silva. Protocol final version and draft manuscript revision, statistical methodology, and data curation: Frederico Monfardini. Original draft manuscript revision, statistical methodology, and data curation: Gustavo Prado dos Santos. Draft manuscript revision and data curation: Bruna dos Santos Sampaio, Fernando Galan Júnior, Flávia Miranda Duarte, Viviane Sant'Anna, Mariane Pereira, Jomenica de Bortoli Livramento, and Denison Alves Pedrosa. Protocol final version and draft manuscript revision, data curation: Diogo Fagundes Moia and Camila Santos Nascimento de Albuquerque. Regulatory documents curation: Luciana Pereira Almeida de Piano. Original protocol, protocol final version, and draft manuscript revision: Ronaldo Soares. Original protocol and draft manuscript revision, methodology, and data curation: Vanessa Teich. Original protocol and draft manuscript revision, methodology, funding acquisition, and conceptualization: Otávio Berwanger. Original draft manuscript revision, supervision, and funding acquisition: Luiz Vicente Rizzo and Gisele Sampaio Silva. Draft manuscript revision: João Brainer Clares de Andrade.

## Funding

The study was designed and led by an academic steering committee and sponsored by a grant from the Brazilian Ministry of Health—PROADI‐SUS (grant number: NUP‐25000.165213/2020‐69), which had no role in study design, data collection, site selection, data management, statistical analysis, or decision to publish the manuscript.

## Ethics Statement

This study received ethical approval at each of the nine participating sites as listed below: Instituto da Criança do Hospital das Clínicas de São Paulo HCFMUSP: CAAE‐27245419.0.2007.0068. Hospital de Clínicas de Porto Alegre: CAAE‐27245419.0.2010.5327. Hospital Infantil Pequeno Príncipe: CAAE‐27245419.0.2002.0097. Hospital de Clínicas da Universidade Estadual de Campinas: CAAE‐27245419.0.2004.5404. Universidade Federal do Estado de São Paulo UNIFESP: CAAE‐27245419.0.2009.5505. Hospital das Clínicas‐Universidade Federal de Minas Gerais UFMG: CAAE‐27245419.0.2005.5149. Hospital Infantil Albert Sabin: CAAE‐27245419.0.2003.5042. Instituto de Puericultura e Pediatria Martagão Gesteira da Universidade Federal do Rio de Janeiro (UFRJ): CAAE‐27245419.0.2001.5264. Hospital Universitário Pedro Ernesto (HUPE): CAAE‐27245419.0.1001.5259.

## Conflicts of Interest

Edmar Zanoteli, Graziela Jorge Polido, Jonas Alex Morales Saute, Adriana Banzzatto Ortega, Marcondes Cavalcante França Junior, Juliana Gurgel Giannetti, and Alexandra Prufer de Queiroz Campos Araújo: Consultant advisory, talks, and principal investigator for Biogen, Novartis, and Roche. Acary Souza Bulle Oliveira, Paulo Victor Sgobbi de Souza, André Luiz Santos Pessoa, Mário Fritsch Toros Neves, and Raquel Tavares Boy da Silva: Consultant advisory and/or talks. Elice Carneiro Batista, Henrique Andrade R. Fonseca, Frederico Monfardini, Gustavo Prado dos Santos, Bruna dos Santos Sampaio, Diogo Fagundes Moia, Fernando Galan Júnior, Flávia Miranda Duarte, Luciana de Piano, Ronaldo Soares, Viviane Sant’ Anna, Mariane Pereira, Jomenica de Bortoli Livramento, Vanessa Teich, Otávio Berwanger, Luiz Vicente Rizzo, João Brainer Clares de Andrade, Gisele Sampaio Silva, Consultant Advisory and/or talks, and work in studies with funding from Biogen and AstraZeneca.

## Supporting information


**Supporting Information: 1** Score on the Expanded Hammersmith Functional Motor Scale (HFMSE) in ambulatory 5q‐SMA type 2 patients considering groups stratified by disease time interval (stratified the data into three disease duration intervals: < 3, 3–7, and > 7 years).
**Supporting Information: 2A:** Score on the Expanded Hammersmith Functional Motor Scale (HFMSE) in ambulatory 5q‐SMA type 3 patients.
*p value = 0.006 adjusted when comparing patients with up to 10 years of time disease (who are currently using nusinersen) versus patients > 10 years of time disease (who are currently using nusinersen), Dunn's test (*p* unadjusted value = 0.001, Kruskal–Wallis test). **p value = 0.019 adjusted when comparing patients with up to 10 years of time disease (who are not using nusinersen) vs. patients > 10 years of time disease (who are not using nusinersen), Dunn's test (unadjusted *p* value = 0.013, Kruskal–Wallis test).
**Supporting Information: 2B:** Score on the Expanded Hammersmith Functional Motor Scale (HFMSE) in ambulatory 5q‐SMA type 3 patients (patients able to walk with support and independently) considering groups stratified by disease time interval.
*p value = 0.010 adjusted when comparing patients with up to 10 years of disease (who are currently using nusinersen) versus patients > 10 years of disease (who are currently using nusinersen), Dunn's test (*p* unadjusted value = 0.001, Kruskal–Wallis test).
**Supporting Information: 2C:** Age at the onset of the first signs and symptoms in 5q‐SMA type 3 patients—Stratification in terms of disease duration.
**p value = 0.001 adjusted when comparing patients with up to 10 years of illness (who are not currently using nusinersen) versus patients > 10 years of illness (who are currently not using nusinersen), Test of Dunn.
**Supporting Information: 3:** Status of treatment with nusinersena of 5q‐SMA type 2 and 3 judicialized patients being monitored in the Brazilian Unified National Health System (SUS).

## Data Availability

The data that support the findings of this study are available on request from the corresponding author. The data are not publicly available due to privacy or ethical restrictions.

## Appendix A

*Instituto da Criança do Hospital das Clínicas de São Paulo FMUSP Team*:

Graziela Jorge Polido, Juliana de Oliveira Achili Ferreira Caires, Luiz Henrique Libardi Silva, and Rodrigo de Holanda Mendonça.

*Hospital de Clínicas de Porto Alegre Team*:

Ana Lúcia Portella Staub, Barbara C. Krug, Fabiana R. C. Machado, Ida Vanessa D. Schwartz, Indara Carmanim Saccilotto, Michele M. Becker, Paulo Dornelles Picon, Tatiane P. S. da Silva, and Viviane Z. Sacharuk.

*Hospital Infantil Pequeno Príncipe Team*:

Caroline Schenknecht, Luiz Felipe de Almeida, Maíra Arrivabene Coelho, and Tauane Gomes da Silva.

*Hospital de Clínicas da Universidade Estadual de Campinas Team*:

Cristina Iwabe, Felipe Franco da Graça, and Maria Fernanda R. Bittar.

*Universidade Federal do Estado de São Paulo UNIFESP Team*:

Francis Favero and Wladimir B. Vieira.

*Hospital das Clínicas‐Universidade Federal de Minas Gerais UFMG Team*:

Ana Carolina Monteiro Lessa de Moura, Bruna Isabela Magalhães, Fabíola Ramos Fernandes, Gabriela Palhares C. Sampaio, Hannelore Teles Rezende Maciel, Josyenne Simão de Lima, Marina Belisário Carvalhais, and Stefania Cotta Doré.

*Hospital Infantil Albert Sabin Team*:

Beatriz Mota, Cristiane Mattos de Oliveira, Raffaela Neves Mont'alverene Napoleão, Rômulo da Nóbrega de Alencar, Tamiris Mariano, Erlane Ribeiro, Jordanne Henrique de Freitas Silva, Marta Maria Sampaio Serrano, and Nayane de Sousa Fernandes.

*Instituto de Puericultura e Pediatria Martagão Gesteira da Universidade Federal do Rio de Janeiro (UFRJ) Team*:

Ana Paula H. Kovacs, Flávia Nardes dos Santos, Jaqueline Almeida Pereira, and Lia Mello Brasil.

*Hospital Universitário Pedro Ernesto (HUPE) Team*:

Cláudia Deolinda L. A. Madureira, Heloisa Viscaino Pereira, and Stella A. E. Pinto dos Santos.
