# Supplementary material for: Clinical Characterization of Patients With 5q Spinal Muscular Atrophy Types 2 and 3 in Brazil: A Cross‐Sectional Observational Study
Source: Clin Genet. 2026 May 8;110(2):172–88. doi: 10.1111/cge.70176 (PMC13327155; doi:10.1111/cge.70176)
Supplement: Supplementary file 1 — Supporting Information: 1 Score on the Expanded Hammersmith Functional Motor Scale (HFMSE) in ambulatory 5q‐SMA type 2 patients considering groups stratified by disease time interval (stratified the data into three disease duration intervals: < 3, 3–7, and > 7 years). Supporting Information: 2A: Score on the Expanded Hammersmith Functional Motor Scale (HFMSE) in ambulatory 5q‐SMA type 3 patients. *p value = 0.006 adjusted when comparing patients with up to 10 years of time disease (who are currently using nusinersen) versus patients > 10 years of time disease (who are currently using nusinersen), Dunn's test (p unadjusted value = 0.001, Kruskal–Wallis test). **p value = 0.019 adjusted when comparing patients with up to 10 years of time disease (who are not using nusinersen) vs. patients > 10 years of time disease (who are not using nusinersen), Dunn's test (unadjusted p value = 0.013, Kruskal–Wallis test). Supporting Information: 2B: Score on the Expanded Hammersmith Functional Motor Scale (HFMSE) in ambulatory 5q‐SMA type 3 patients (patients able to walk with support and independently) considering groups stratified by disease time interval. *p value = 0.010 adjusted when comparing patients with up to 10 years of disease (who are currently using nusinersen) versus patients > 10 years of disease (who are currently using nusinersen), Dunn's test (p unadjusted value = 0.001, Kruskal–Wallis test). Supporting Information: 2C: Age at the onset of the first signs and symptoms in 5q‐SMA type 3 patients—Stratification in terms of disease duration. **p value = 0.001 adjusted when comparing patients with up to 10 years of illness (who are not currently using nusinersen) versus patients > 10 years of illness (who are currently not using nusinersen), Test of Dunn. Supporting Information: 3: Status of treatment with nusinersena of 5q‐SMA type 2 and 3 judicialized patients being monitored in the Brazilian Unified National Health System (SUS). [file CGE-110-172-s001.docx]

**Supplementary material**

**Supplementary material 1:** Score on the Expandend Hammersmith Functional Motor Scale (HFMSE) in ambulatory 5q-SMA type 2 patients considering groups stratified by disease time interval (stratified the data into three disease duration intervals: < 3 years, 3 to 7 years, and > 7 years).

|  | | **5q SMA type 2** | | | | | | | | | | | | | | | |
| --- | --- | --- | --- | --- | --- | --- | --- | --- | --- | --- | --- | --- | --- | --- | --- | --- | --- |
|  | | **< 3 years** | | | | **3 to 7 years** | | | | **> 7 years** | | | | **Total** | | | |
| Use of nusinersen? | | Yes | | No | | Yes | | No | | Yes | | No | | Yes | | No | |
|  |  | (N=10) | | (N=4) | | (N=13) | | (N=12) | | (N=16) | | (N=21) | | (N=39) | | (N=37) | |
| **HFMSE** | |  | |  | |  | |  | |  | |  | |  | |  | |
| Mean (SD) | | 23.9 (18.4) | | 16.5 (17.7) | | 21.8 (16.6) | | 13.5 (10.7) | | 9.23 (9.33) | | 8.27 (8.84) | | 17.2 (15.5) | | 10.6 (10.0) | |
| Median [Q1, Q3] | | 17.00 [9.00, 42.50] | | 16.50 [10.25, 22.75] | | 14.00 [11.00, 30.00] | | 11.00 [9.00, 15.00] | | 5.00 [4.00, 10.00] | | 5.00 [3.00, 11.00] | | 11.00 [5.75, 24.50] | | 6.00 [3.00, 14.00] | |
| [min, max] | | [3.00, 44.00] | | [4.00, 29.00] | | [6.00, 53.00] | | [2.00, 34.00] | | [0.00, 27.00] | | [0.00, 30.00] | | [0.00, 53.00] | | [0.00, 34.00] | |
| Missing | | 3 (30.0%) | | 2 (50.0%) | | 1 (7.7%) | | 4 (33.3%) | | 3 (18.8%) | | 6 (28.6%) | | 7 (17.9%) | | 12 (32.4%) | |
|  | |  | |  | |  | |  | |  | |  | |  | |  | |
|  | | **5q SMA type 2** | | | | | | | | | | | | | | | |
|  | | **< 3 years** | | | | **3 to 7 years** | | | | **> 7 years** | | | | **Total** | | | |
| Use of nusinersen? | | Yes | | No | | Yes | | No | | Yes | | No | | Yes | | No | |
|  |  | (N=10) | | (N=4) | | (N=13) | | (N=12) | | (N=16) | | (N=21) | | (N=39) | | (N=37) | |
| **HFMSE** | |  | |  | |  | |  | |  | |  | |  | |  | |
| Mean (SD) | | 23.9 (18.4) | | 16.5 (17.7) | | 21.8 (16.6) | | 13.5 (10.7) | | 9.23 (9.33) | | 8.27 (8.84) | | 17.2 (15.5) | | 10.6 (10.0) | |
| Median [Q1, Q3] | | 17.00 [9.00, 42.50] | | 16.50 [10.25, 22.75] | | 14.00 [11.00, 30.00] | | 11.00 [9.00, 15.00] | | 5.00 [4.00, 10.00] | | 5.00 [3.00, 11.00] | | 11.00 [5.75, 24.50] | | 6.00 [3.00, 14.00] | |
| [min, max] | | [3.00, 44.00] | | [4.00, 29.00] | | [6.00, 53.00] | | [2.00, 34.00] | | [0.00, 27.00] | | [0.00, 30.00] | | [0.00, 53.00] | | [0.00, 34.00] | |
| Missing | | 3 (30.0%) | | 2 (50.0%) | | 1 (7.7%) | | 4 (33.3%) | | 3 (18.8%) | | 6 (28.6%) | | 7 (17.9%) | | 12 (32.4%) | |

**Supplementary material 2A:** Score on the Expandend Hammersmith Functional Motor Scale (HFMSE) in ambulatory 5q-SMA type 3 patients.

|  | **5q-SMA type 3** | | | | | |
| --- | --- | --- | --- | --- | --- | --- |
|  | **0 to 10 years** | | **> 10 years** | | **Total** | |
| Use of nusinersen? | Yes | No | Yes | No | Yes | No |
|  | (N=13) | (N=19) | (N=8) | (N=39) | (N=21) | (N=58) |
| **HFMSE** |  |  |  |  |  |  |
| Mean (SD) | 47.3 (12.5) | 42.6 (14.8) | 19.1 (13.4)* | 28.6 (17.2)** | 36.9 (18.7) | 33.6 (17.6) |
| Median [Q1, Q3] | 49.00 [39.50, 57.25] | 46.00 [33.00, 56.00] | 18.00 [9.50, 28.00] | 28.50 [12.00, 40.00] | 39.00 [25.50, 55.50] | 36.00 [18.50, 46.50] |
| [min, max] | [25.00, 64.00] | [14.00, 64.00] | [2.00, 39.00] | [1.00, 61.00] | [2.00, 64.00] | [1.00, 64.00] |
| Missing | 1 (7.7%) | 2 (10.5%) | 1 (12.5%) | 9 (23.1%) | 2 (9.5%) | 11 (19.0%) |

*p value= 0.006 adjusted when comparing patients with up to 10 years of time disease (who are currently using nusinersen) vs patients >10 years of time disease (who are currently using nusinersen), Dunn's test (p unadjusted value= 0.001 Kruskal-Wallis test). **p value= 0.019 adjusted when comparing patients with up to 10 years of time disease (who are not using nusinersen) vs patients >10 years of time disease (who are not using nusinersen), Dunn's test (unadjusted p value= 0.013 Kruskal-Wallis test).

**Supplementary material 2B:** Score on the Expandend Hammersmith Functional Motor Scale (HFMSE) in ambulatory 5q-SMA type 3 patients (patients able to walk with support and independently) considering groups stratified by disease time interval.

|  | **5q SMA type 3 (ambulant)** | | | | | |
| --- | --- | --- | --- | --- | --- | --- |
|  | **0 to 10 years** | | **> 10 years** | | **Total** | |
| Use of nusinersen? | Yes | No | Yes | No | Yes | No |
|  | (N=9) | (N=15) | (N=5) | (N=20) | (N=14) | (N=35) |
| **HFMSE** |  |  |  |  |  |  |
| Mean (SD) | 48.3 (10.7) | 41.2 (14.9) | 15.6 (14.6)* | 35.1 (14.4) | 36.6 (20.0) | 37.7 (14.7) |
| Median [Q1, Q3] | 55.00 [41.00, 57.00] | 43.00 [30.00, 54.75] | 14.00 [5.00, 18.00] | 38.00 [27.00, 41.50] | 40.00 [21.50, 55.75] | 40.00 [29.00, 47.00] |
| [min, max] | [32.00, 59.00] | [14.00, 62.00] | [2.00, 39.00] | [7.00, 61.00] | [2.00, 59.00] | [7.00, 62.00] |
| Missing | 0 (0%) | 1 (6.7%) | 0 (0%) | 1 (5.0%) | 0 (0%) | 2 (5.7%) |

*p value= 0.010 adjusted when comparing patients with up to 10 years of disease (who are currently using nusinersen) vs patients >10 years of disease (who are currently using nusinersen), Dunn's test (p unadjusted value= 0.001 Kruskal-Wallis test).

**Supplementary material 2C:** Age at the onset of the first signs and symptoms in 5q-SMA type 3 patients – Stratification in terms of disease duration.

|  | **5q SMA type 3** | | | | | |
| --- | --- | --- | --- | --- | --- | --- |
|  | **0 to 10 years** | | **> 10 years** | | **Total** | |
| Use of nusinersen? | Yes | No | Yes | No | Yes | No |
|  | (N=13) | (N=19) | (N=8) | (N=39) | (N=21) | (N=58) |
| **Age of first symptoms onset (years)** |  |  |  |  |  |  |
| Mean (SD) | 2.92 (4.32) | 2.30 (2.46) | 4.95 (4.33) | 6.82 (5.35)** | 3.69 (4.34) | 5.34 (5.06) |
| Median [Q1, Q3] | 2.00 [1.00, 3.00] | 1.50 [1.00, 2.00] | 4.00 [1.75, 6.50] | 5.00 [2.00, 13.00] | 2.00 [1.00, 4.00] | 2.50 [1.50, 8.75] |
| [min, max] | [0.50, 17.00] | [0.75, 11.00] | [0.58, 12.00] | [1.00, 15.00] | [0.50, 17.00] | [0.75, 15.00] |
| Missing | 0 (0%) | 0 (0%) | 0 (0%) | 0 (0%) | 0 (0%) | 0 (0%) |

**p value= 0.001 adjusted when comparing patients with up to 10 years of illness (who are not currently using nusinersen) vs patients >10 years of illness (who are currently not using nusinersen), Test of Dunn.

**Supplementary material 3:** Status of treatment with nusinersena of 5q-SMA type 2 and 3 judicialized patients being monitored in the Brazilian Unified National Health System (SUS).

|  | 5q SMA Type 2 (N=39) | 5q SMA Type 3 (N=21) | Total (N=60) |
| --- | --- | --- | --- |
| Nusinersena treatment phase |  |  |  |
| Starting doses | 1 (2.6%) | 2 (9.5%) | 3 (5.0%) |
| Maintenance dose | 34 (87.2%) | 14 (66.7%) | 48 (80.0%) |
| Fourth Dose | 4 (10.3%) | 5 (23.8%) | 9 (15.0%) |
| Use of support techniques for the application of Nusinersena | | | |
| None | 14 (35.9%) | 12 (57.1%) | 26 (43.3%) |
| Fluroscopy/Radioscopy | 0 (0.0%) | 1 (4.8%) | 1 (1.6%) |
| Tomography | 3 (7.7%) | 1 (4.8%) | 4 (6.6%) |
| Unknown | 1 (2.5%) | 1 (4.8%) | 2 (3.3%) |
| Others | 21 (53.9%) | 6 (28.5%) | 27 (45.0%) |
| Report of occurrence of adverse events during infusion | | | |
| No | 36 (92.3%) | 21 (100.0%) | 57 (95.0%) |
| Unknown | 3 (7.7%) | 0 (0.0%) | 3 (5.0%) |
| Report of post-lumbar puncture syndrome presented by the patient | | | |
| Yes | 5 (12.8%) | 3 (14.3%) | 8 (13.3%) |
| No | 34 (87.2%) | 17 (80.9%) | 51 (85.0%) |
| Unknown | 0 (0.0%) | 1 (4.8%) | 1 (1.6%) |
